# Supplementary figures and images for: Approximation to the Distribution of Fitness Effects across Functional Categories in Human Segregating Polymorphisms
Source: PLoS Genet. 2014 Nov 6;10(11):e1004697. doi: 10.1371/journal.pgen.1004697 (PMC4222666; doi:10.1371/journal.pgen.1004697)

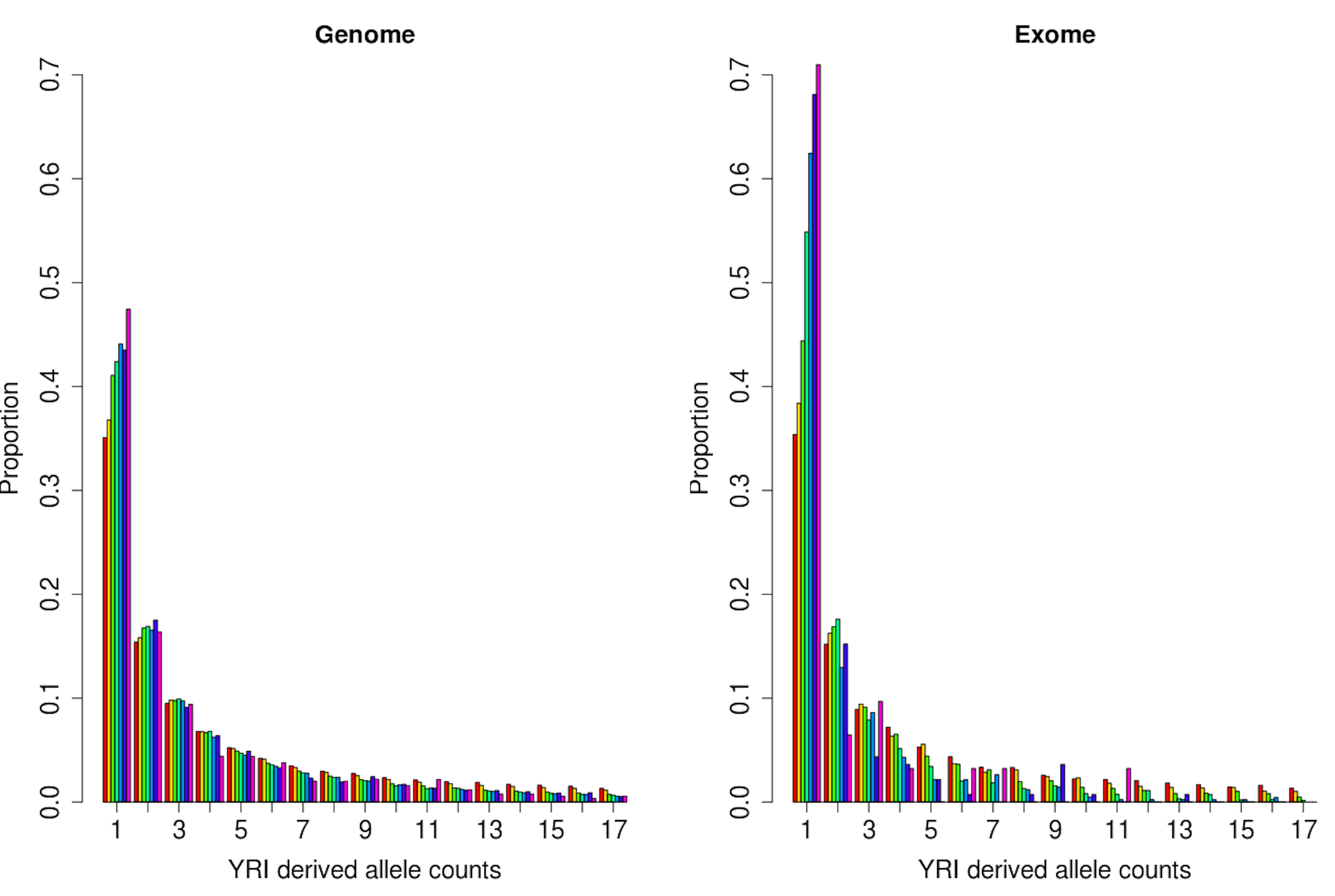

Supplement: Figure S1 — Observed SFS for sites under different C-score bins using the Complete Genomics YRI data, for all autosomes in the genome (left) and the exome (right). Note that the spectrum gets more skewed towards singletons with increasing C-scores, likely reflecting the action of negative selection on deleterious mutations. (TIFF) [file pgen.1004697.s001.tiff]

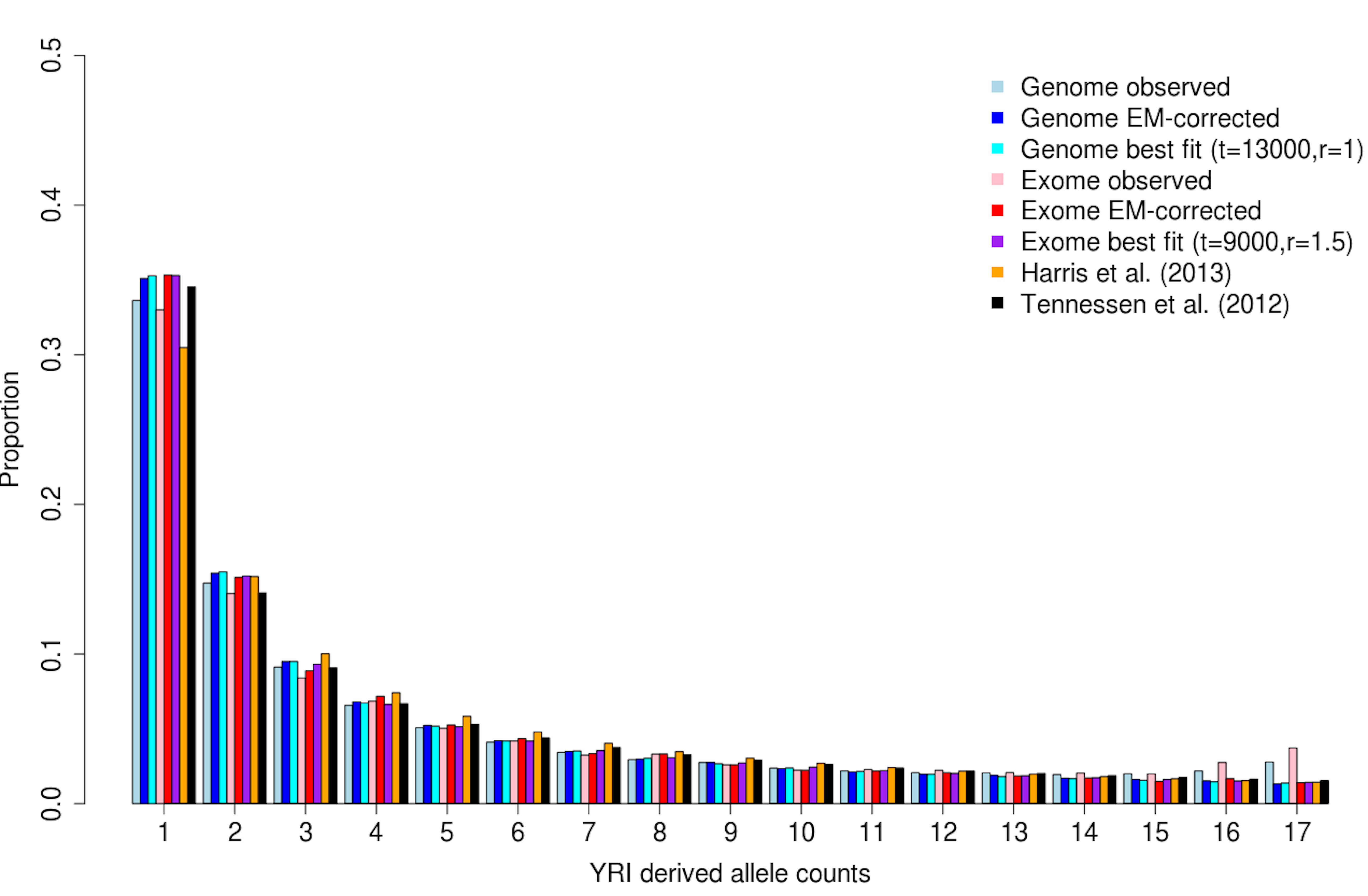

Supplement: Figure S2 — Observed SFS of YRI Complete Genomics data for sites with C = 0. The full SFS was corrected for ancestral state misidentification using an EM algorithm and fit to different models of neutral evolution. We show results for both the genome and the exome. (TIFF) [file pgen.1004697.s002.tiff]

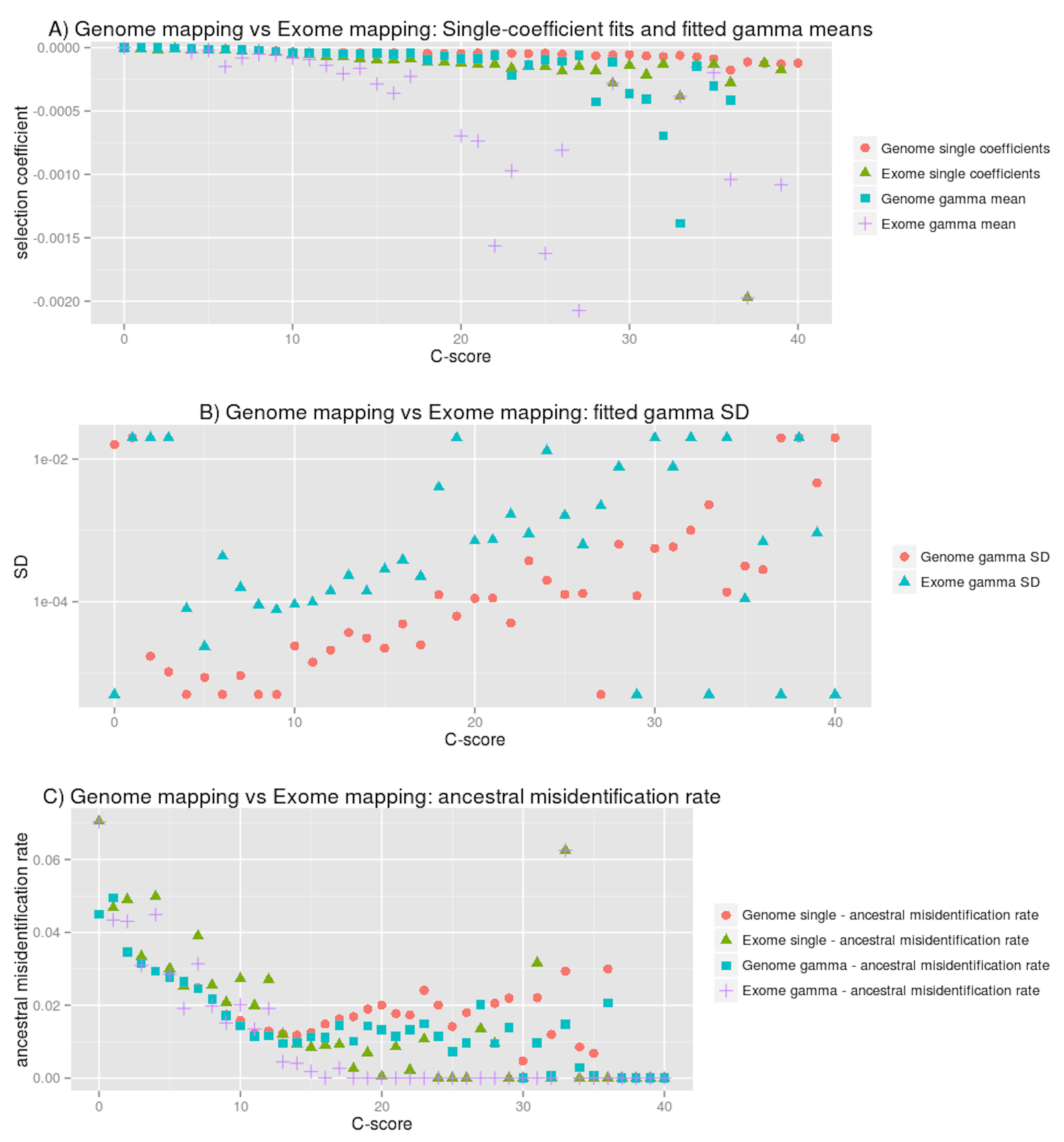

Supplement: Figure S3 — Features of fitted single-coefficient and gamma distributions. A) Fitted single coefficients and means of fitted gamma distributions for each C-score bin, using genome-wide or exome-wide polymorphisms. B) Standard deviation of fitted gamma distributions for each bin. C) Ancestral misidentification rate obtained from an EM algorithm used to jointly fit the data and infer this rate at each bin. SD = standard deviation. (TIFF) [file pgen.1004697.s003.tiff]

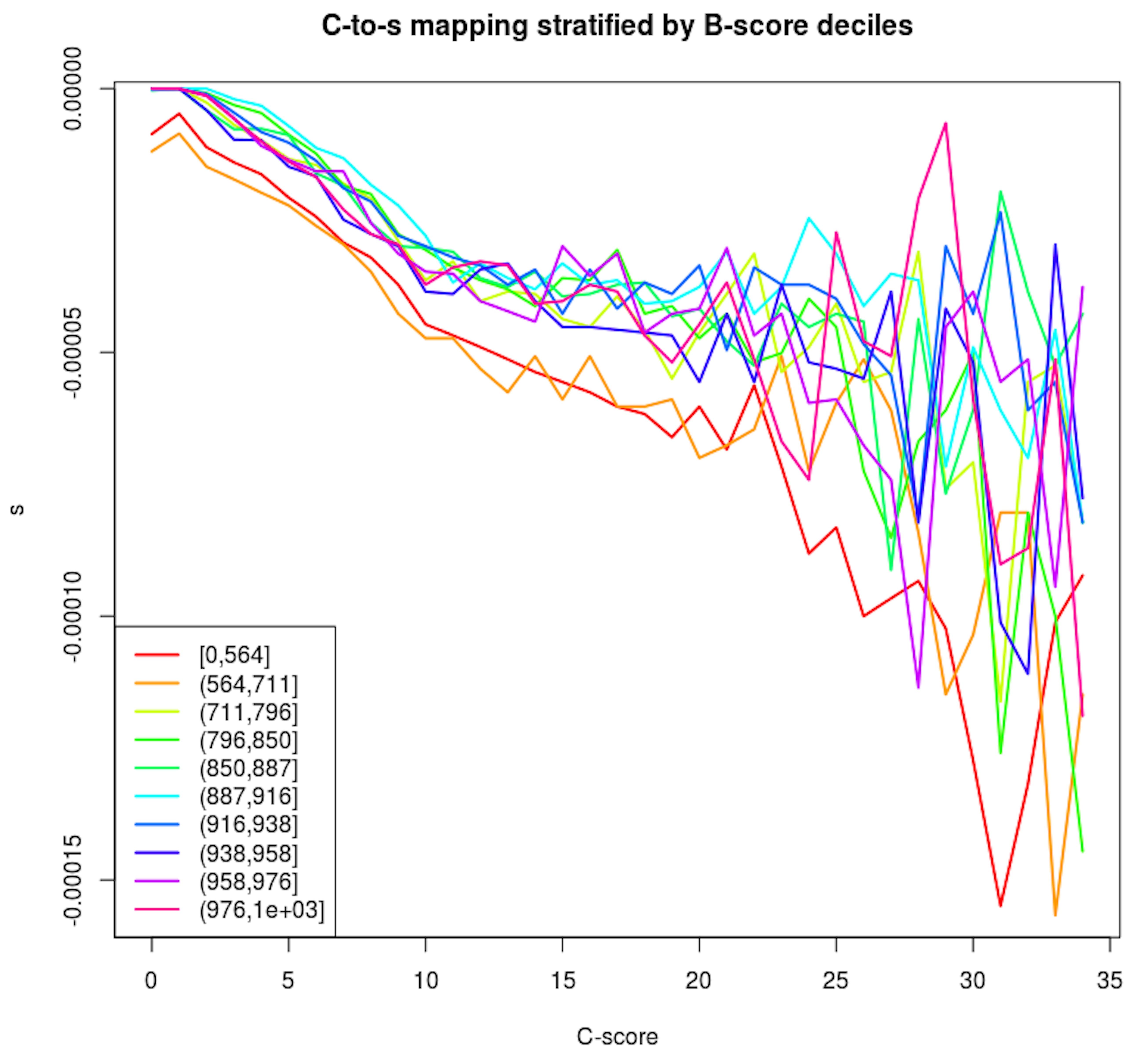

Supplement: Figure S4 — C-to-s mapping stratified by B-score deciles. We partitioned the genome by deciles of B-scores [35], which reflect levels of background selection. Then, we recomputed the demographic fitting and C-to-s mapping for each decile. (TIFF) [file pgen.1004697.s004.tiff]

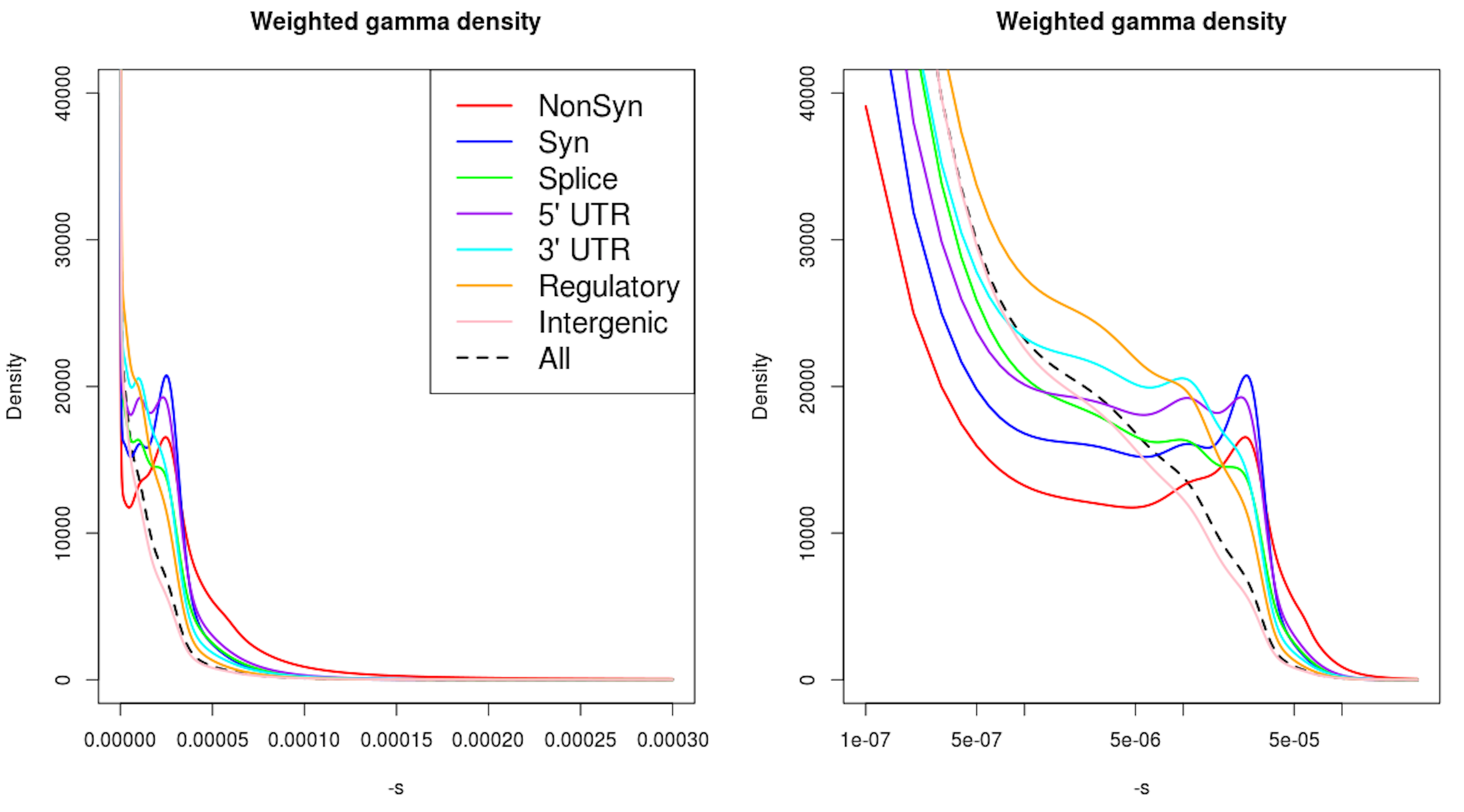

Supplement: Figure S5 — Inferred DFEs for different classes of polymorphisms obtained from gamma distribution fittings of each C-score bin. The plot shows, for each category, a weighted sum of gamma distributions, where each C-score bin contributes its corresponding genome-wide best-fitting gamma distribution in proportion to the number of polymorphisms present at that bin. (TIFF) [file pgen.1004697.s005.tiff]

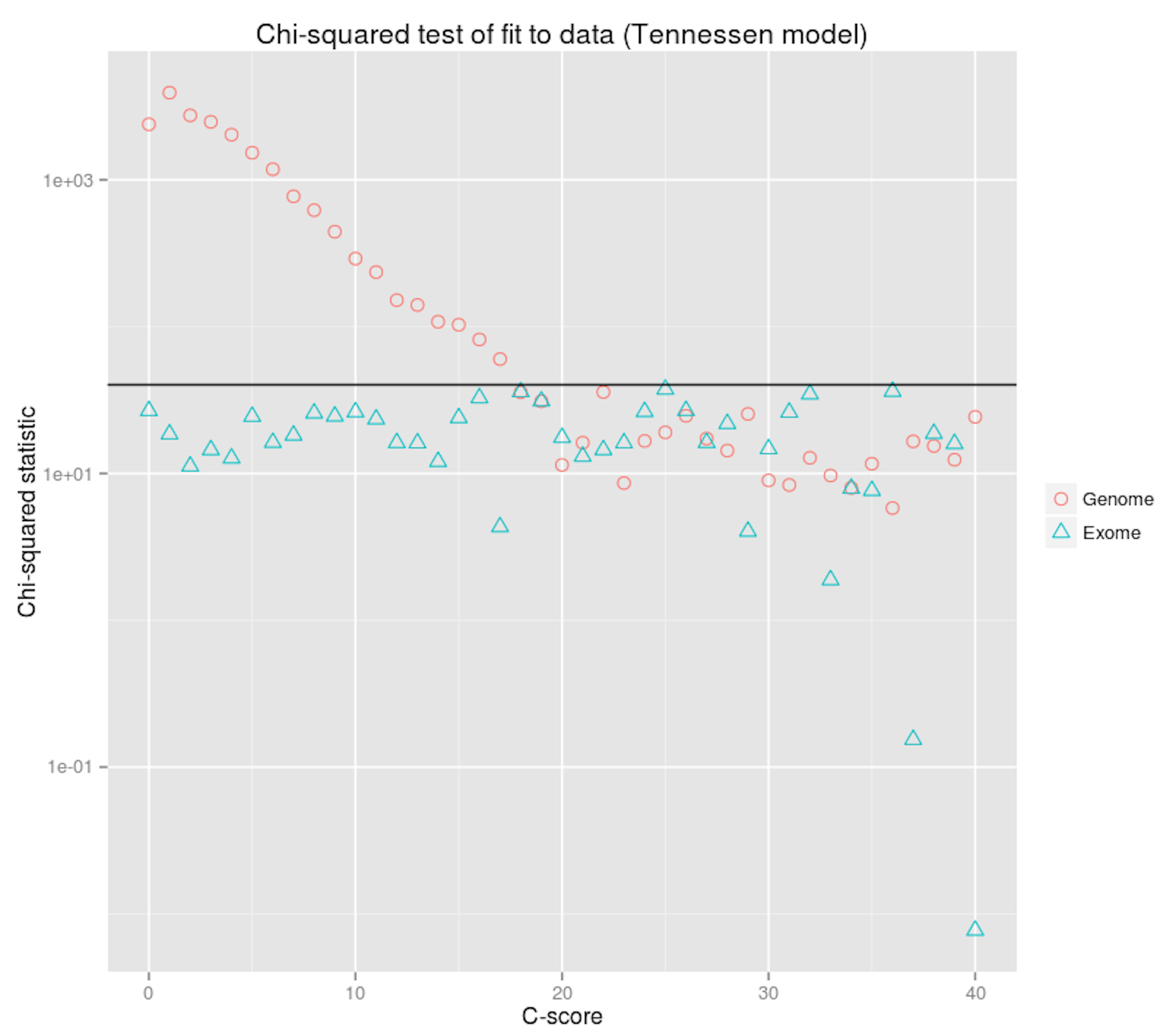

Supplement: Figure S6 — Chi-squared test of the fit of the single-coefficient model to the data at each bin, using the human demography inferred from ref. [33]. As with simpler models (Figure 1.F), we observe significant scores at low C-score bins when using the genome-wide data, but not the exome-wide data. (TIFF) [file pgen.1004697.s006.tiff]

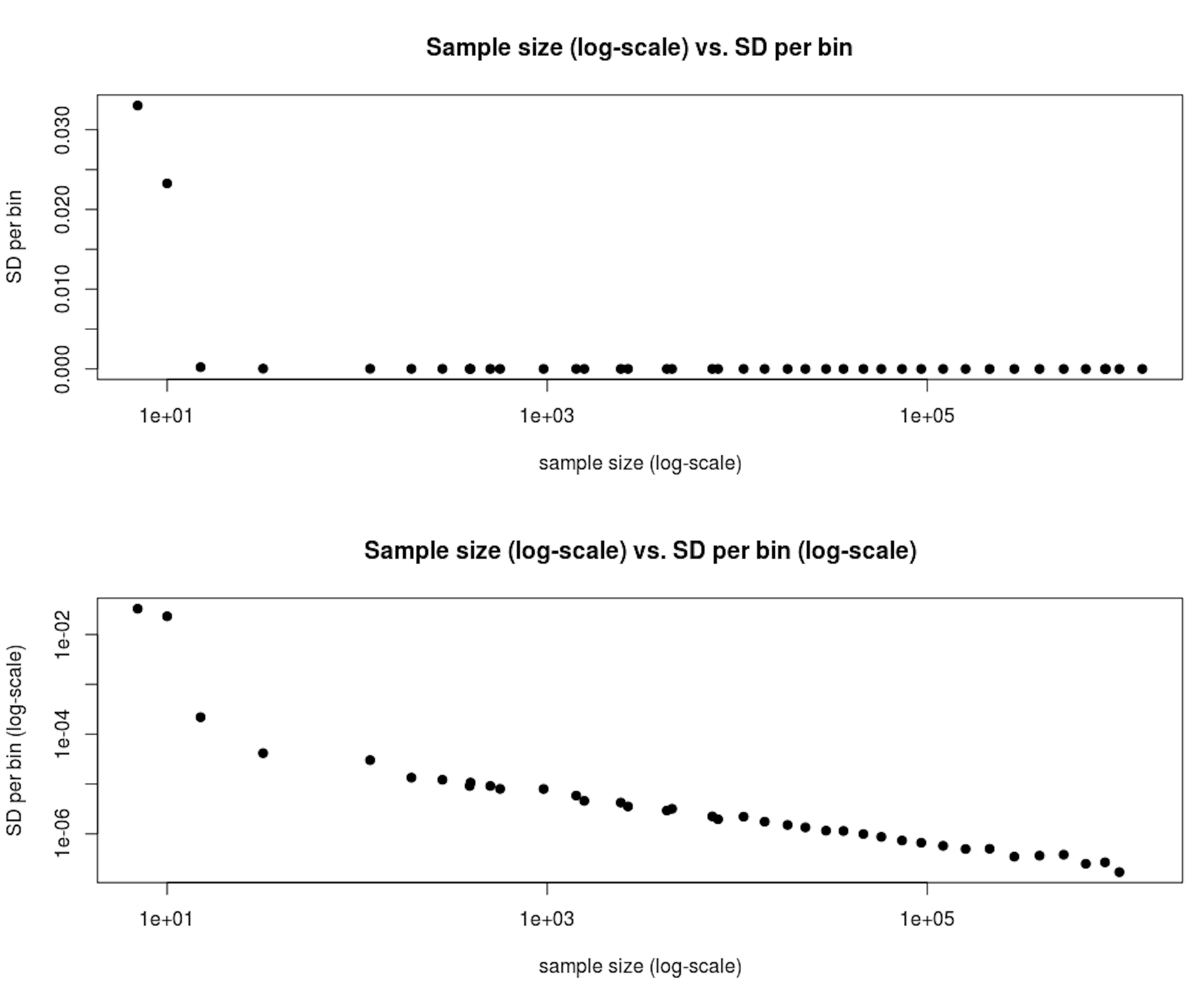

Supplement: Figure S7 — Comparison between the size of each C-score bin and the standard deviation of single-coefficient fits obtained from 100 bootstraps of the data within each bin. Top panel: Standard deviation per C-score bin plotted as a function of sample size per bin (log-scale). Bottom panel: Same plot but with the y-axis on a log-scale. (TIFF) [file pgen.1004697.s007.tiff]

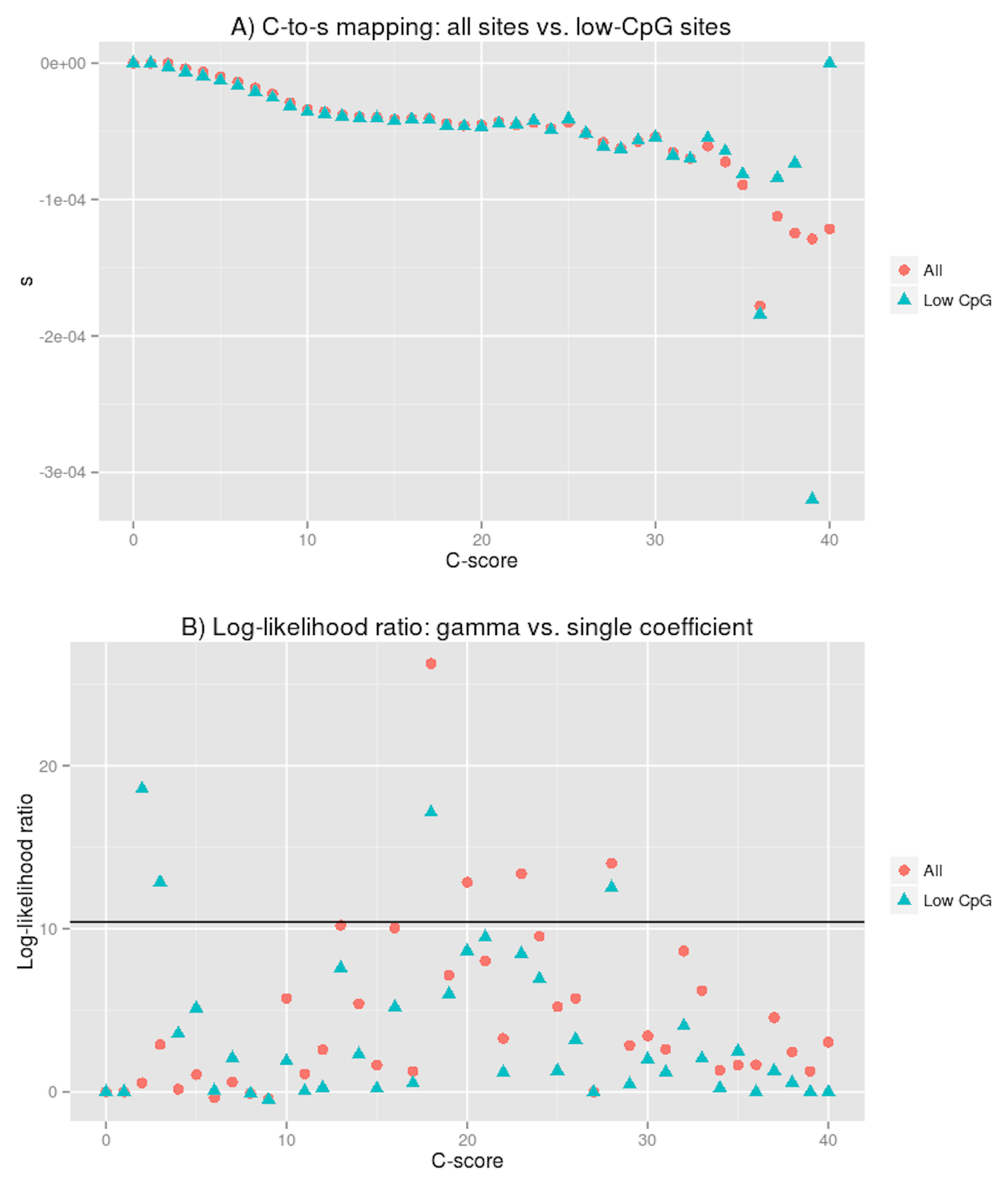

Supplement: Figure S8 — Mapping of sites with low CpG density. A) We filtered for sites with low CpG density, such that the proportion of CpG sites in a +/− 75 bp window around each site was <0.05, and then recomputed the C-to-s mapping. B) We also repeated the gamma fitting and calculated a likelihood ratio test of the gamma model against the single-coefficient model at each C-score bin. (TIFF) [file pgen.1004697.s008.tiff]

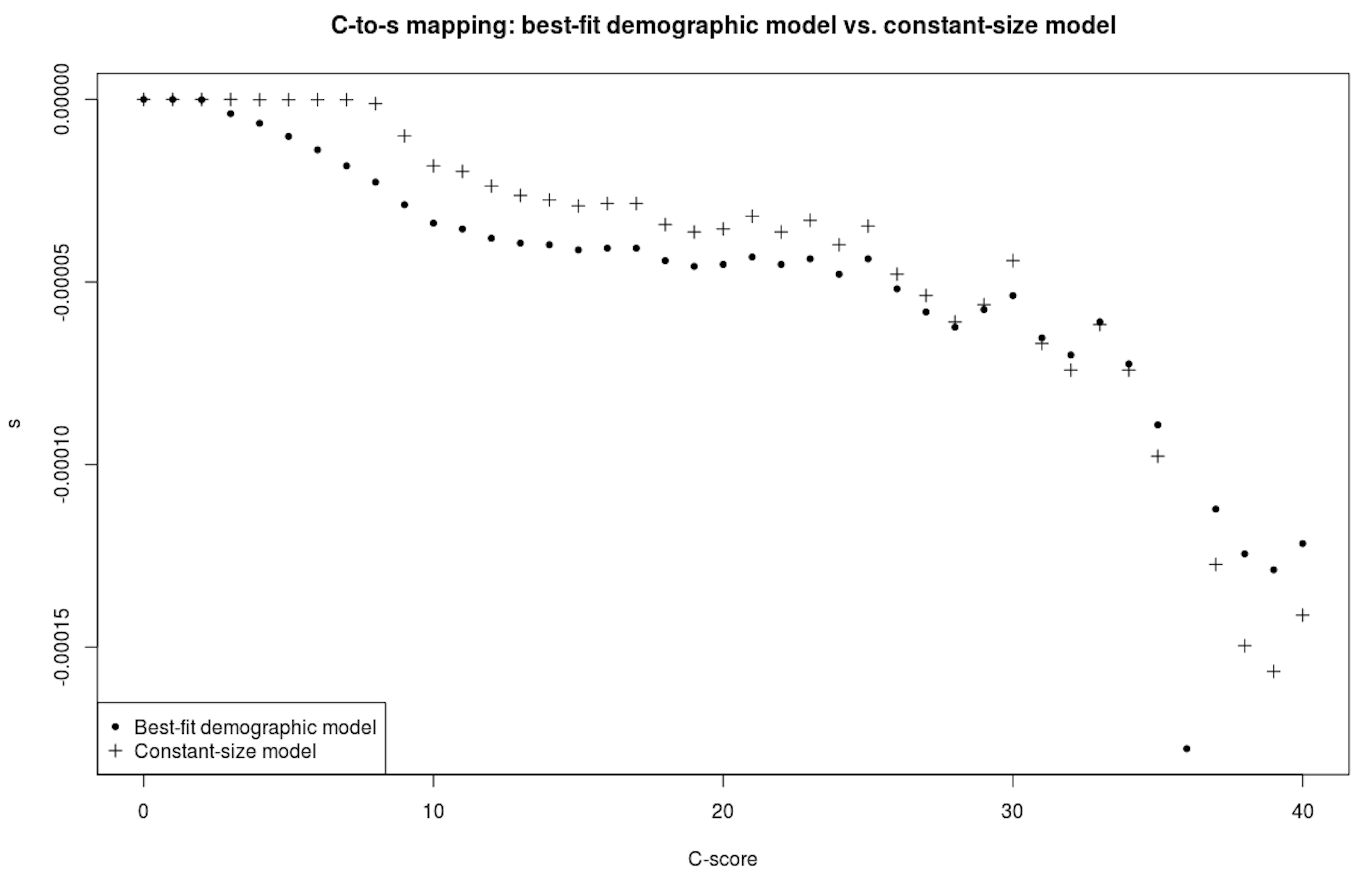

Supplement: Figure S9 — Comparison between a C-to-s mapping using the best-fit demographic model and a constant-size model. The best-fit model is exponential growth with = 13,000 and r = 1. (TIFF) [file pgen.1004697.s009.tiff]

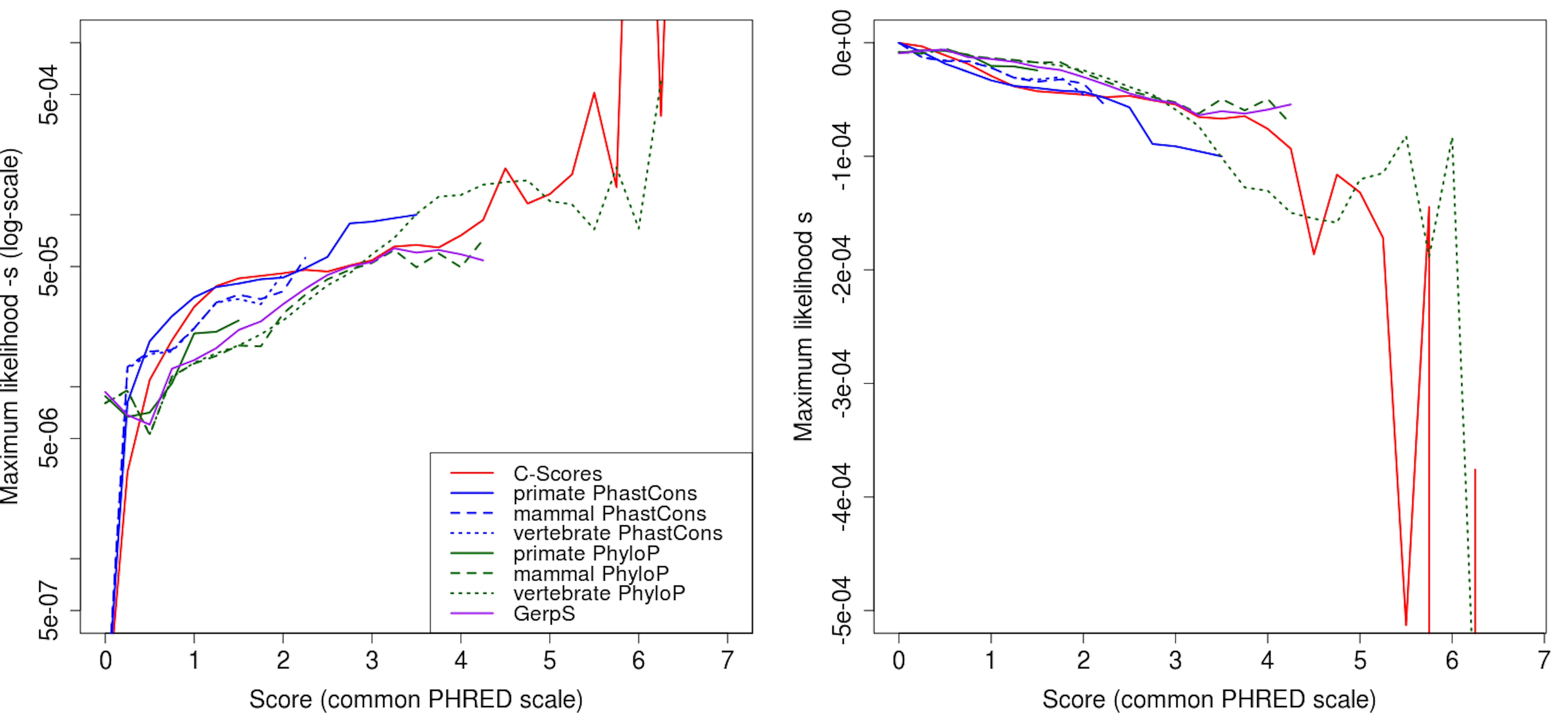

Supplement: Figure S10 — Maximum likelihood mapping of different types of scores to a selection coefficient scale, excluding bins mapped to neutrality, using the Complete Genomics data. Before mapping, scores were re-scaled on a common PHRED scale, by converting each score to –log10(p) where is the probability of observing a change as or more disruptive/conserved (based on that particular score scale) among all polymorphic YRI sites. Some scores extend over larger PHRED scores than others because they have a finer stratification (smaller number of sites with tied scores). The wide fluctuations to the right of the figures are due to the small number of sites per bin at highly deleterious bins. (TIFF) [file pgen.1004697.s010.tiff]

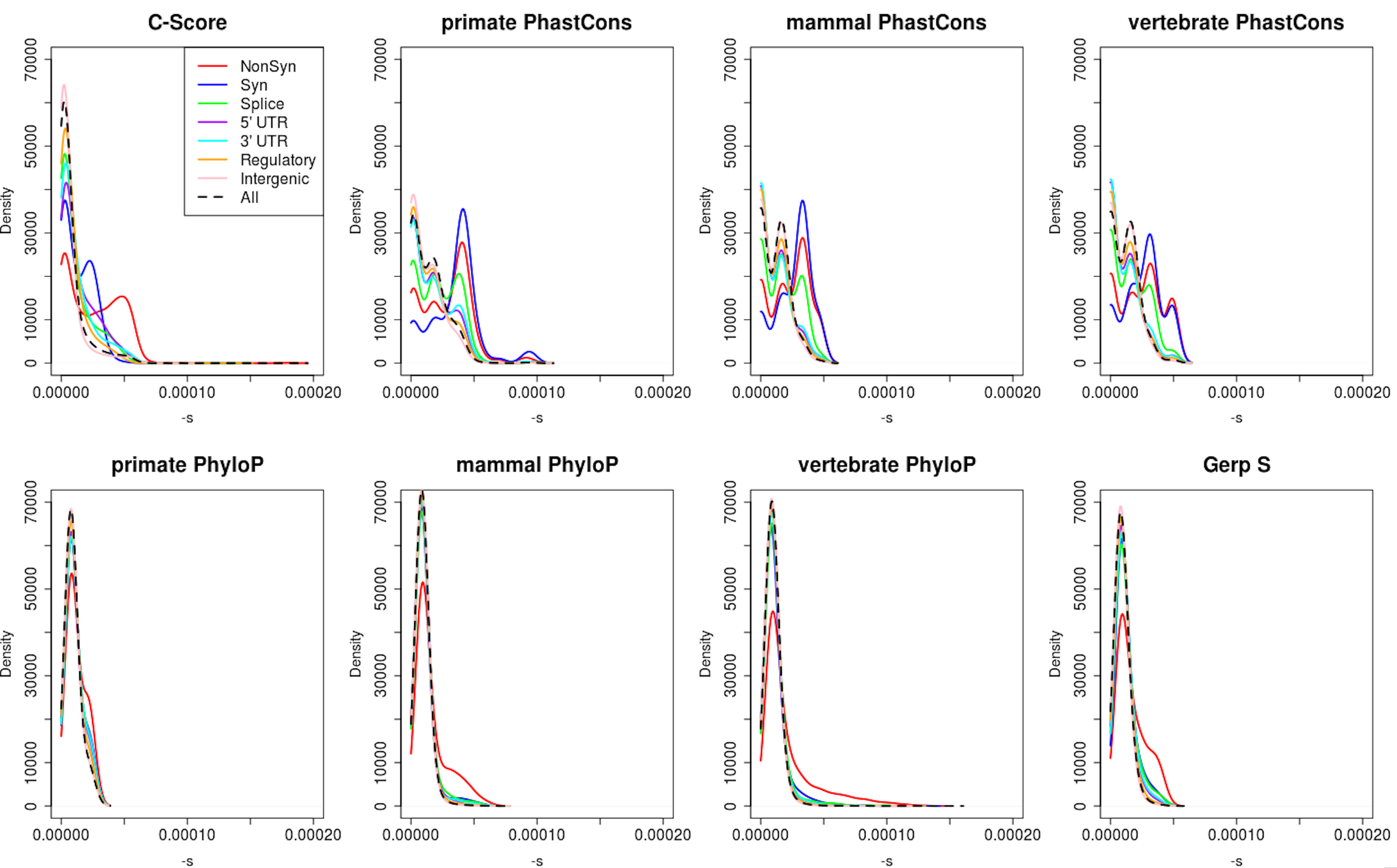

Supplement: Figure S11 — Distribution of fitness effects at different types of polymorphisms in Yoruba, using different types of conservation scores for mapping (smoothing bandwidth = 0.000005). We only mapped sites with PHRED-scaled scores ≤5, because the mappings become erratic for higher values, due to the small number of sites per bin (Figure S10). (TIFF) [file pgen.1004697.s011.tiff]

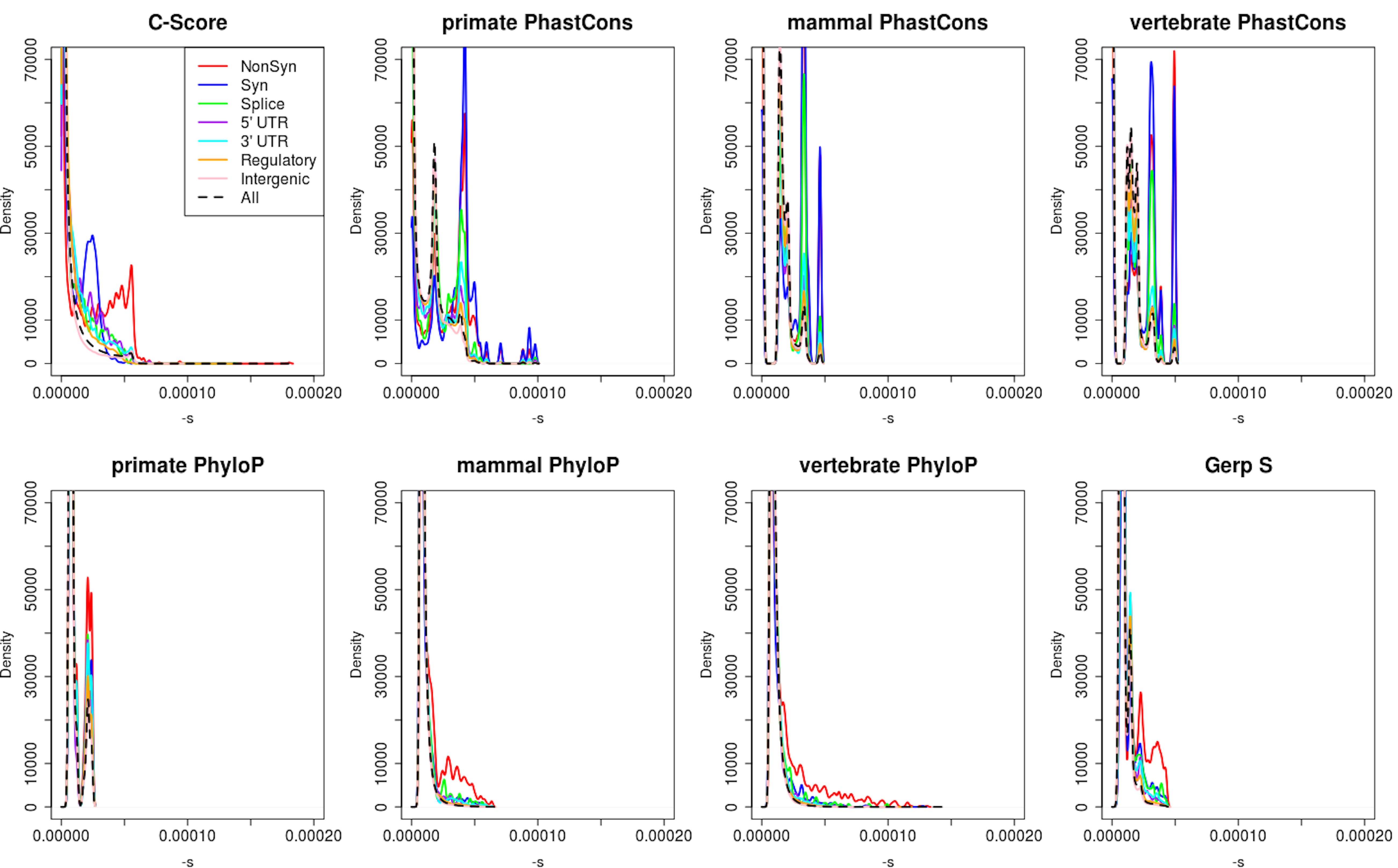

Supplement: Figure S12 — Distribution of fitness effects at different classes of polymorphisms in Yoruba, using different types of conservation scores for mapping (smoothing bandwidth = 0.000001). We only mapped sites with PHRED-scaled scores ≤5, because the mappings become erratic for higher values, due to the small number of sites per bin (Figure S10). (TIFF) [file pgen.1004697.s012.tiff]

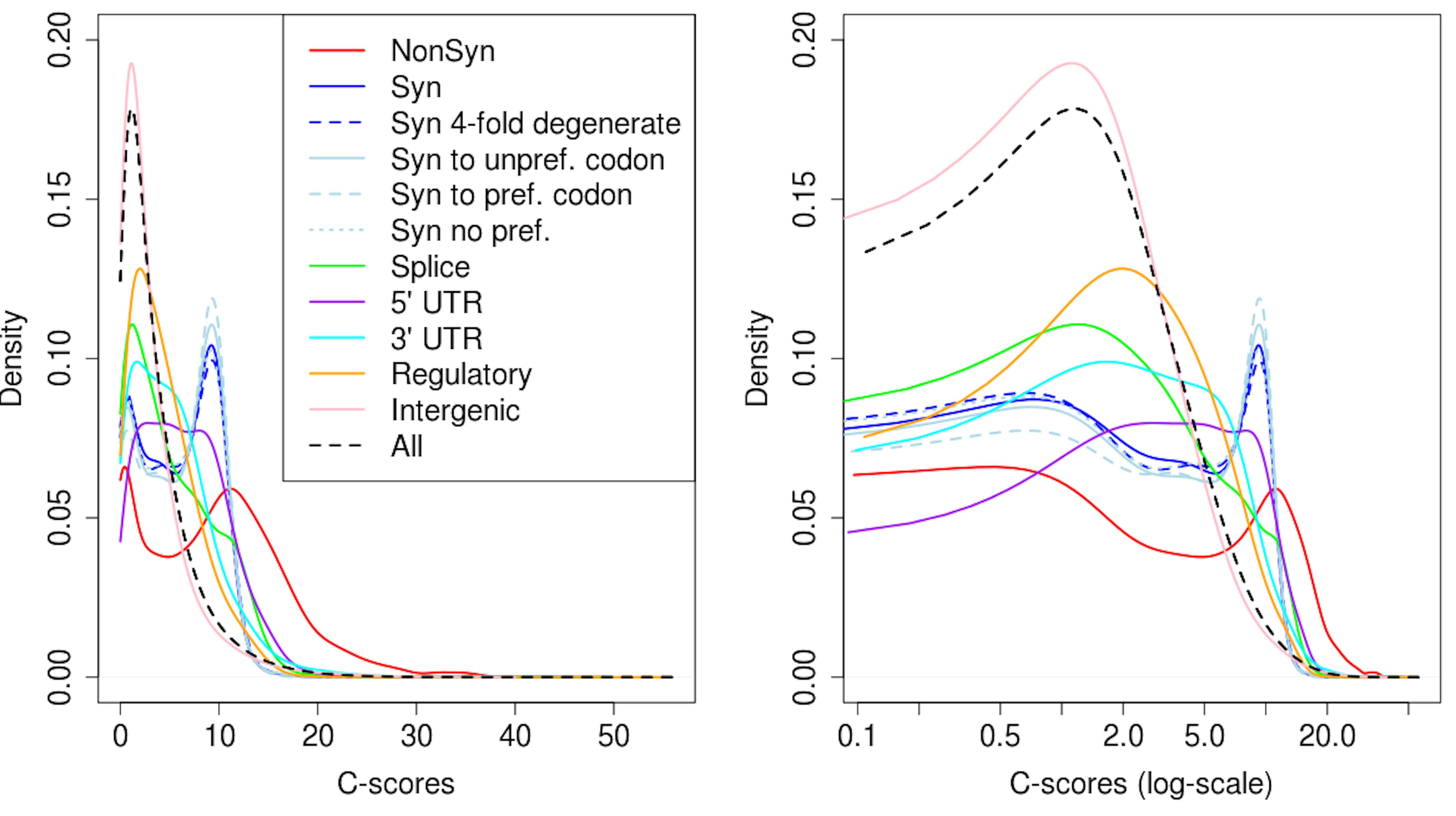

Supplement: Figure S13 — Distribution of unmapped C-scores among YRI polymorphisms, partitioned by the genomic consequence of the mutated site. Consequences were determined using the Ensembl Variant Effect Predictor (v.2.5). Codon and degeneracy information was obtained from snpEff. NonSyn = nonsynonymous. Syn = synonymous. Syn to unpref. codon = synonymous change from a preferred to an unpreferred codon. Syn to pref. codon = synonymous change from an unpreferred to a preferred codon. Syn no pref. = synonymous change from an unpreferred codon to a codon that is also unpreferred. Splice = splice site. (TIFF) [file pgen.1004697.s013.tiff]

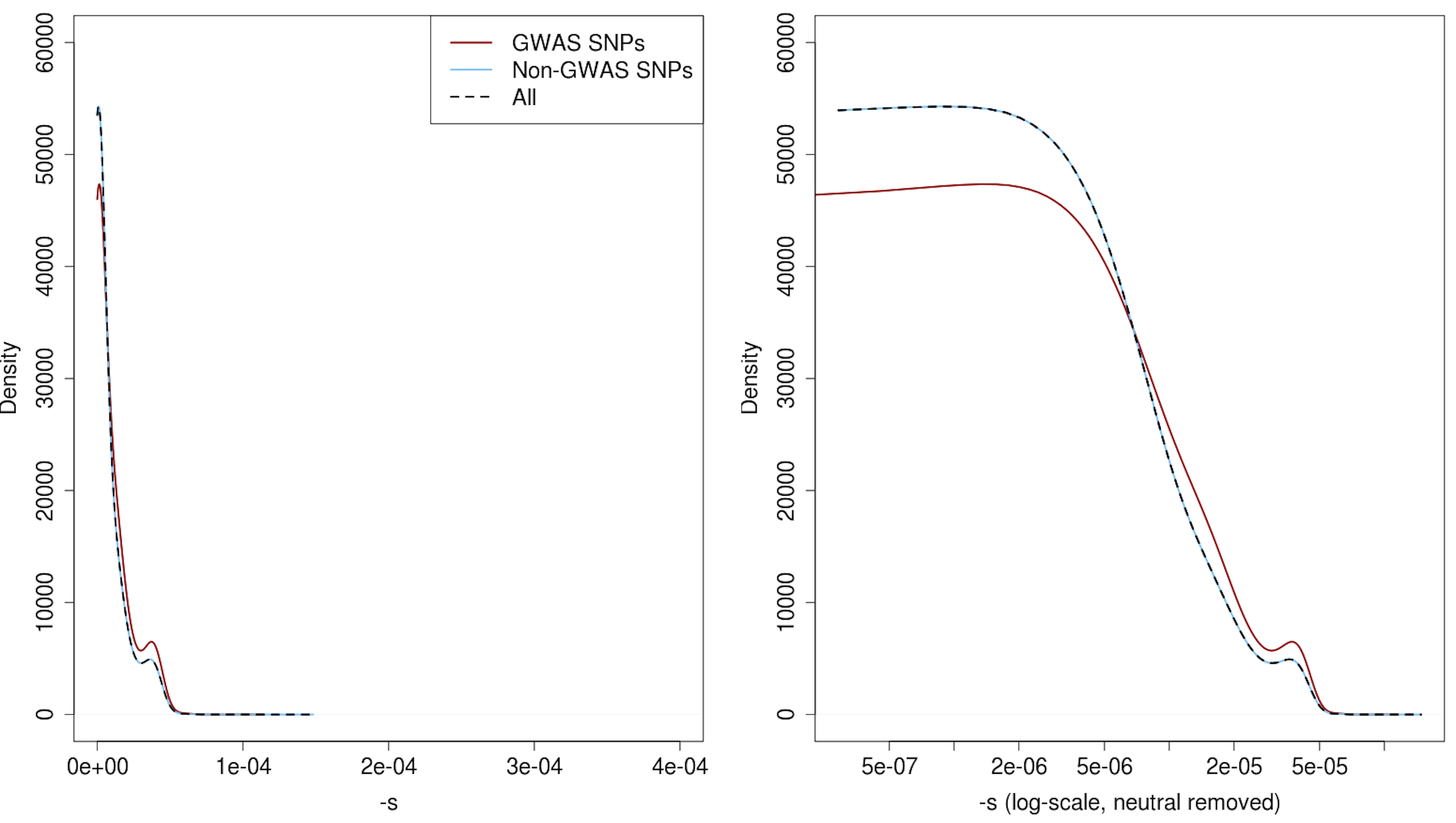

Supplement: Figure S14 — Distribution of fitness effects among YRI polymorphisms, partitioned by whether the SNPs are found in the GWAS database or not. The right panel shows a zoomed-in version of the same distributions after removing neutral polymorphisms and log-scaling the x-axis. (TIFF) [file pgen.1004697.s014.tiff]

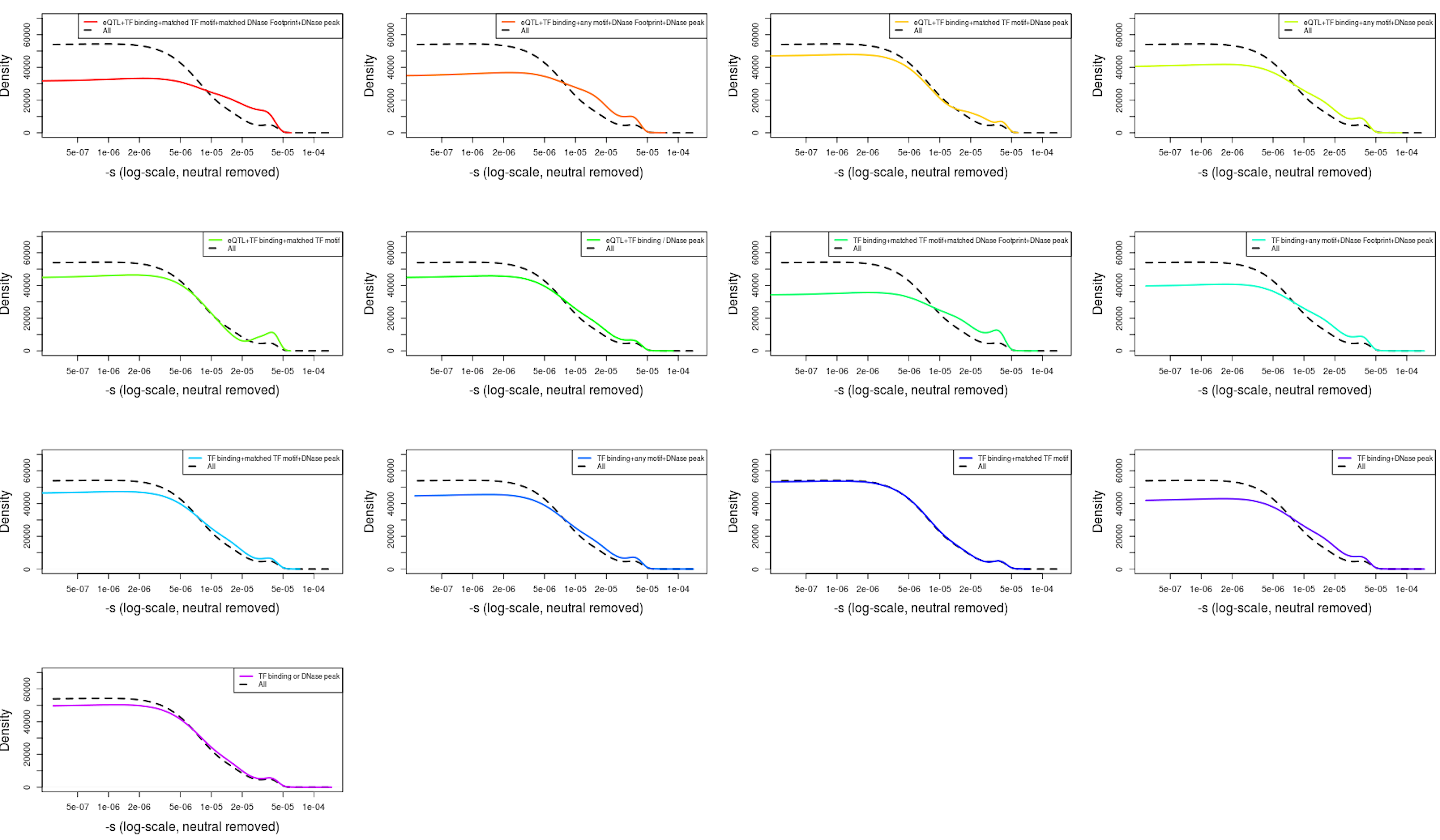

Supplement: Figure S15 — Distribution of fitness effects among different types of RegulomeDB regulatory YRI polymorphisms, obtained from various ENCODE assays. The black dashed line corresponds to the distribution of all YRI SNPs. (TIFF) [file pgen.1004697.s015.tiff]

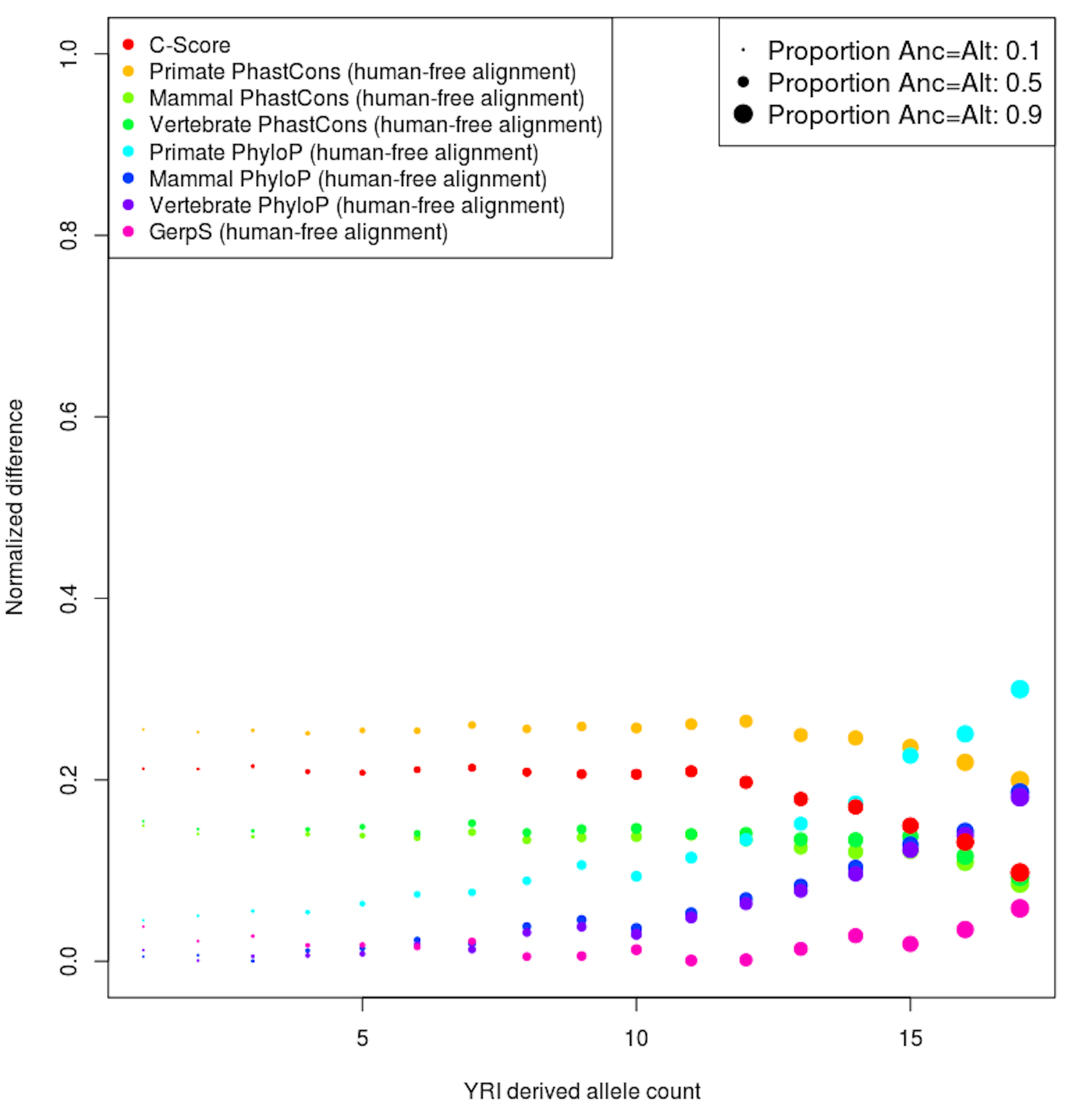

Supplement: Figure S16 — Comparison of reference/alternative bias observed in C-scores and human-free conservation scores. For each score, we computed the absolute difference in means of scores at sites where reference = ancestral and at sites where reference = derived, divided by the total standard deviation at both types of sites, and plotted as a function of the number of derived alleles. The size of each circle denotes the proportion of sites where alternative = ancestral at each derived allele bin. (TIFF) [file pgen.1004697.s016.tiff]
