# Supplementary material for: Approximation to the Distribution of Fitness Effects across Functional Categories in Human Segregating Polymorphisms
Source: PLoS Genet. 2014 Nov 6;10(11):e1004697. doi: 10.1371/journal.pgen.1004697 (PMC4222666; doi:10.1371/journal.pgen.1004697)
Supplement: Table S1 — Characteristics of fitness effect distributions estimated for YRI SNPs classified by different genomic consequence categories, RegulomeDB categories and Segway categories, using the exome-wide C-to-s mapping. We show quantiles of selection coefficients, the log base 10 of the mean selection coefficient and the log base 10 of the standard deviation of coefficients in each category. (PDF) [file pgen.1004697.s017.pdf]

| Category | n | $\lvert s\rvert < 10^{-5}$ | $\lvert s\rvert \geq 10^{-5}$ | $\lvert s\rvert \geq 5{*}10^{-5}$ | $\lvert s\rvert \geq 10^{-4}$ | $\log_{10}(\overline{-s})$ | $\log_{10}(SD(-s))$ |
|---|---|---|---|---|---|---|---|
| All | 9065398 | 40.51% | 59.49% | 5.87% | 0.92% | -4.79 | -4.7 |
| Nonsynonymous | 32522 | 17.26% | 82.74% | 45.87% | 11.52% | -4.28 | -4.35 |
| Synonymous | 30630 | 22% | 78% | 9.77% | 0.06% | -4.61 | -4.74 |
| Synonymous 4-fold degenerate | 16895 | 22.79% | 77.21% | 10.70% | 0.03% | -4.61 | -4.73 |
| Synonymous to unpreferred codon | 12498 | 21.54% | 78.46% | 10.52% | 0.10% | -4.59 | -4.73 |
| Synonymous to preferred codon | 4574 | 19.52% | 80.48% | 10.08% | 0% | -4.59 | -4.75 |
| Synonymous no preference change | 11844 | 22.55% | 77.45% | 9.58% | 0.03% | -4.61 | -4.74 |
| Splice site | 10122 | 25.43% | 74.57% | 16.08% | 0.42% | -4.6 | -4.63 |
| 5' UTR | 23125 | 13.95% | 86.05% | 18.65% | 0.88% | -4.52 | -4.55 |
| 3' UTR | 81605 | 20.98% | 79.02% | 11.94% | 1.60% | -4.6 | -4.58 |
| Regulatory | 864769 | 23.20% | 76.80% | 7.42% | 0.14% | -4.7 | -4.75 |
| Intergenic | 3544204 | 44.21% | 55.79% | 5.35% | 1.12% | -4.82 | -4.69 |
| GWAS | 8693 | 30.16% | 69.84% | 8.24% | 1.69% | -4.7 | -4.64 |
| eQTL+TF binding+matched TF motif+matched DNase Footprint+DNase peak | 240 | 14.58% | 85.42% | 11.67% | 0.83% | -4.6 | -4.7 |
| eQTL+TF binding+any motif+DNase Footprint+DNase peak | 1965 | 18.78% | 81.22% | 9.97% | 0.51% | -4.64 | -4.69 |
| eQTL+TF binding+matched TF motif+DNase peak | 62 | 25.81% | 74.19% | 8.06% | 1.61% | -4.69 | -4.65 |
| eQTL+TF binding+any motif+DNase peak | 1263 | 22.33% | 77.67% | 9.11% | 0.55% | -4.67 | -4.7 |
| eQTL+TF binding+matched TF motif | 44 | 31.82% | 68.18% | 13.64% | 2.27% | -4.62 | -4.56 |
| eQTL+TF binding / DNase peak | 26610 | 27.75% | 72.25% | 7.16% | 0.86% | -4.71 | -4.7 |
| TF binding+matched TF motif+matched DNase Footprint+DNase peak | 12411 | 17.94% | 82.06% | 13.42% | 0.54% | -4.6 | -4.65 |
| TF binding+any motif+DNase Footprint+DNase peak | 120642 | 22.76% | 77.24% | 9.62% | 0.82% | -4.66 | -4.66 |
| TF binding+matched TF motif+DNase peak | 5355 | 30.51% | 69.49% | 7.79% | 1.05% | -4.72 | -4.68 |
| TF binding+any motif+DNase peak | 96905 | 28.49% | 71.51% | 8.61% | 1.29% | -4.69 | -4.64 |
| TF binding+matched TF motif | 4359 | 38.63% | 61.37% | 6.33% | 0.99% | -4.78 | -4.7 |
| TF binding+DNase peak | 418548 | 24.64% | 75.36% | 8.15% | 0.87% | -4.68 | -4.68 |
| TF binding or DNase peak | 1625195 | 33.43% | 66.57% | 7.02% | 1.32% | -4.74 | -4.66 |
| C0 - CTCF (strong) | 20813 | 18.32% | 81.68% | 6.75% | 0.15% | -4.69 | -4.77 |
| C1 - CTCF (weak) | 61946 | 27.65% | 72.35% | 5.21% | 0.46% | -4.75 | -4.77 |
| D - dead zone | 873933 | 52.91% | 47.09% | 4.24% | 0.65% | -4.89 | -4.75 |
| E/GM - enhancer/gene middle | 70593 | 23.64% | 76.36% | 7.94% | 0.67% | -4.68 | -4.7 |
| F0 - FAIRE only | 1177948 | 36.88% | 63.12% | 6.41% | 1.04% | -4.76 | -4.69 |
| F1- FAIRE only | 1481766 | 41.02% | 58.98% | 5.94% | 1.03% | -4.79 | -4.69 |
| GE0 - gene body (end) | 413390 | 33.96% | 66.04% | 8.03% | 1.09% | -4.72 | -4.66 |
| GE1 - gene body (end) | 163365 | 28.37% | 71.63% | 8.06% | 1.03% | -4.7 | -4.67 |
| GE2 - gene body (end) | 54261 | 32.46% | 67.54% | 6.38% | 0.94% | -4.74 | -4.66 |
| GM0 - gene body (middle) | 130000 | 27.23% | 72.77% | 7.27% | 0.82% | -4.71 | -4.7 |
| GM1 - gene body (middle) | 101426 | 24.46% | 75.54% | 6.44% | 0.62% | -4.71 | -4.73 |
| GS - gene body (start) | 54940 | 10.49% | 89.51% | 11.35% | 0.45% | -4.58 | -4.7 |
| H3K9me1 | 457492 | 62.34% | 37.66% | 2.70% | 0.27% | -4.98 | -4.85 |
| L0 - low zone | 673274 | 52.14% | 47.86% | 4.02% | 0.67% | -4.89 | -4.76 |
| L1 - low zone | 725876 | 33.64% | 66.36% | 6.32% | 1.24% | -4.75 | -4.68 |
| N/A | 53354 | 35.18% | 64.82% | 0.87% | 0.07% | -4.96 | -5.09 |
| R0 - repression | 716710 | 42.48% | 57.52% | 6.18% | 1.11% | -4.79 | -4.68 |
| R1 - repression | 335824 | 32.41% | 67.59% | 6.33% | 1.01% | -4.75 | -4.69 |
| R2 - repression | 447655 | 34.45% | 65.55% | 7.83% | 1.37% | -4.73 | -4.65 |
| R3 - repression | 433156 | 41.40% | 58.60% | 5.10% | 0.85% | -4.81 | -4.72 |
| R4 - repression | 205971 | 41.10% | 58.90% | 6.35% | 0.80% | -4.78 | -4.7 |
| R5 - repression | 297152 | 31.70% | 68.30% | 5.15% | 0.66% | -4.77 | -4.74 |
| TF0 - transcription factor activity | 346083 | 35.10% | 64.90% | 4.81% | 0.69% | -4.79 | -4.75 |
| TF1 - transcription factor activity | 327695 | 45.07% | 54.93% | 4.85% | 0.65% | -4.83 | -4.74 |
| TF2 - transcription factor activity | 127366 | 33.95% | 66.05% | 5.69% | 0.82% | -4.76 | -4.68 |
| TSS - transcription start site | 25144 | 5.01% | 94.99% | 21.84% | 0.89% | -4.46 | -4.6 |
